# Development of software enabling Chinese medicine-based precision treatment for osteoporosis at the gene and pathway levels

**DOI:** 10.1186/s13020-022-00596-6

**Published:** 2022-04-15

**Authors:** Jinyu Li, Guiyu Feng, Haoyang He, Haolin Wang, Jia Tang, Aiqing Han, Xiaohong Mu, Weifeng Zhu

**Affiliations:** 1 Department of Orthopedic, Dongzhimen Hospital, Beijing University of Chinese Medicine, No. 5 Haihaicang, Dongcheng District, Beijing, 100007 China; 2 Key Laboratory of Chinese Internal Medicine of Ministry of Education and Beijing, Dongzhimen Hospital, Beijing University of Chinese Medicine, Beijing, China; 3 Key Laboratory of Modern Preparation of TCM, Jiangxi University of Traditional Chinese Medicine, Ministry of Education, 1688 Meiling Avenue, Xinjian District, Nanchang, 330004 Jiangxi China; 4 Dongzhimen Hospital, Beijing University of Chinese Medicine, No. 5 Haihaicang, Dongcheng District, Beijing, 100007 China; 5 School of Management, Beijing University of Chinese Medicine, 11 North Third Ring East Road, Chaoyang District, Beijing, 100105 China

**Keywords:** Osteoporosis, Traditional Chinese medicine, Python programming language, Data mining, Gene and pathway levels

## Abstract

**Background:**

Precision medicine aims to address the demand for precise therapy at the gene and pathway levels. We aimed to design software to allow precise treatment of osteoporosis (OP) with Chinese medicines (CMs) at the gene and pathway levels.

**Methods:**

PubMed, EMBASE, Cochrane Library, China National Knowledge Infrastructure (CNKI), China Science and Technology Journal Database (VIP database), and the Wanfang database were searched to identify studies treating osteoporosis with CMs. The TCMSP was used to identify bioactive ingredients and related genes for each CM. Gene expression omnibus (GEO) database and the limma package were used to identify differentially expressed genes in osteoporosis. Perl software was used to identify the shared genes between the bioactive components in CM and osteoporosis. R packages and bioconductor packages were used to define the target relationship between shared genes and their related pathways. Third-party Python libraries were used to write program codes. Pyinstaller library was used to create an executable program file.

**Results:**

Data mining: a total of 164 CMs were included, but Drynariae Rhizoma (gusuibu) was used to present this process. We obtained 44 precise relationships among the bioactive ingredients of Drynariae Rhizoma, shared genes, and pathways. Python programming: we developed the software to show the precise relationship among bioactive ingredients, shared genes, and pathways for each CM, including Drynariae Rhizoma.

**Conclusions:**

This study could increase the precision of CM, and could provide a valuable and convenient software for searching precise relationships among bioactive ingredients, shared genes, and pathways.

**Supplementary Information:**

The online version contains supplementary material available at 10.1186/s13020-022-00596-6.

## Background

Osteoporosis, a systemic skeletal disease, is defined by an overall deterioration of bone mass and bone microstructure [23], consequently increasing bone fragility and susceptibility to fractures [4]. With a reduction in hip bone mineral density (BMD), hip fractures (prototypical osteoporotic fractures) occur more frequently [17]. Hip fractures, which are characterized by pain and an inability to bear weight, invariably require surgical fixation. Hip fractures are associated with a greater reduction in functional status, substantial direct medical costs, poor quality of life, and even a high risk of short-term mortality. Notably, approximately 2.7 million hip fractures occurred in 2010 worldwide. One study estimated that 51% of hip fractures (with a total of 1,364,717 patients; 264,162 men and 1,100,555 women) were preventable if osteoporosis (defined as a femoral neck T-score ≤ − 2.5 SD) could be detected and treated early [21].

Traditional Chinese medicine (TCM) has become increasingly popular because of its effectiveness and fewer side effects. Natural Chinese medicine, with its effects on the growth and development of skeletal tissue [25, 28], has been widely and effectively used to treat bone loss and bone diseases, such as bone fractures, rheumatism, and osteoporosis [9, 19, 27]. Several studies have shown that TCM can promote bone formation, attenuate imbalanced bone resorption, improve bone mineral density, increase biomechanical properties, and reduce bone microstructural degradation [9, 27, 29], thus exerting anabolic and anticatabolic effects in the treatment of osteoporosis. The results of in vitro experiments indicated that TCM could promote the proliferation and survival of osteoblasts and induce osteoblastic differentiation of bone mesenchymal stem cells (MSCs). However, considering TCM as a useful therapy for osteoporosis at the gene and pathway levels requires further investigation.

Precision medicine, a movement in clinical practice, aims to develop treatments that specifically address the demand for precise therapy at the gene and pathway levels [3]. In the United States, the precision medicine market is predicted to increase from $39 billion in 2015 to more than $87 billion by 2023. This phenomenon indicates that there will be a sharp increase in the demand for precision medicine technologies. Gene therapy has been investigated as a possible treatment for osteoporosis. Delivery of osteogenic genes to precise anatomical locations has shown great potential for bone regeneration and fracture healing. Small interfering RNA (siRNA) therapy has shown tremendous potential in preclinical studies of osteoporosis, and has been widely investigated as a potential therapeutic approach [24]. A siRNA-mediated knock-down of a nuclear factor of active T cells (NFATc1), a transcription factor involved in osteoclast formation, can inhibit LPS-induced osteoclast generation in murine monocyte RAW264.7 cells [6]. A knockdown of PPAR-γ or adiponectin receptor 1 in osteoblastic cells from a liposome-based siRNA transfection prevented the downregulation of mRNA expression of Runx related transcription factor 2 (Runx2) [16]. siRNA delivery targeting of RANK to both RAW264.7 and primary bone marrow cell cultures produced a short-term repression of RANK expression without off-target effects, and significantly inhibited both osteoclast formation and bone resorption [30].

**Fig. 1:**
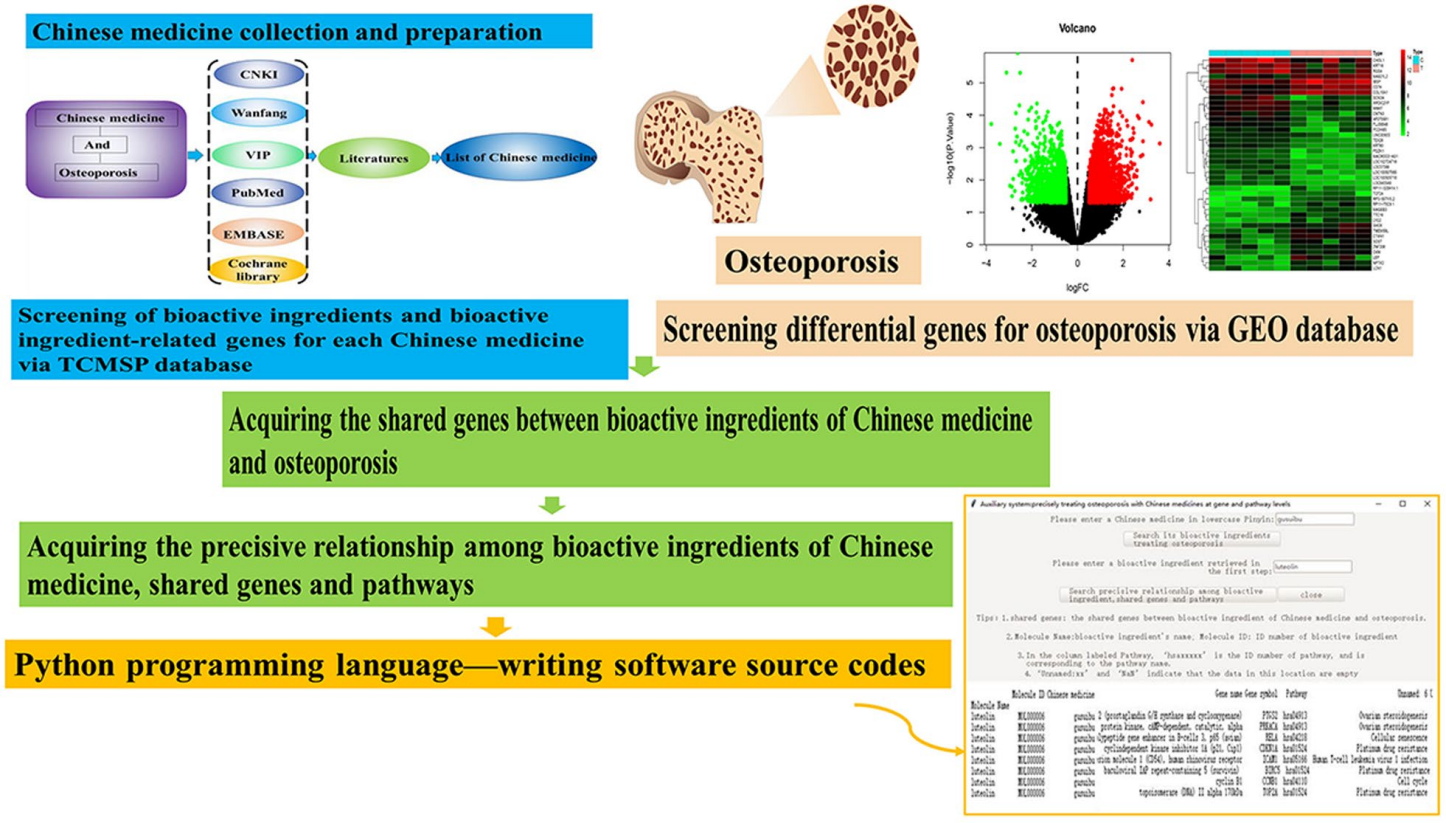
The technical strategy of the current study

In this context, we aimed to obtain ‘precision TCM’ to facilitate the precise treatment of osteoporosis with CMs at the gene and pathway levels. As precision medicine moves forward, new strategies require carriers to express them [1]. The Python programming language is commonly used to create freely available open-source software. Therefore, in this study, we designed a precision TCM-related software using the Python programming language to achieve the precise treatment of osteoporosis with bioactive ingredients of CMs at the gene and pathway levels. The technical strategy used in this study is illustrated in Fig. 1.

## Methods

### Data mining—basic work with the Python Programming

#### Collection and preparation of CM

##### Database search strategies

Six databases including, PubMed, EMBASE, Cochrane library, China National Knowledge Infrastructure (CNKI), China Science and Technology Journal Database (VIP database), and Wanfang database were searched from their inception till August 18th, 2021. All studies published in English and Chinese were searched. The detailed search strategy for PubMed is shown in Appendix A.

##### Study selection and CM collection

The retrieved literature from electronic databases was imported into NoteExpress to delete duplicates. Two authors (Tang and Wang) independently screened the titles, abstracts, and full texts of the remaining studies to identify eligible studies according to the inclusion and exclusion criteria. The inclusion and exclusion criteria for original studies is as follows: (1) Patients with osteoporosis were included. (2) Interventions involving Chinese medicines were included. (3) Any study design was included. (4) Literatures unabling to obtain Chinese medicines were excluded. The same authors independently extracted Chinese medicines from the eligible studies. Any disagreement was submitted to a third author (Jinyu Li) and resolved by his judgment.

#### Screening of bioactive ingredients and related genes for each CM

##### Identification of bioactive components for each CM

The Traditional Chinese Medicine Systems Pharmacology Database and Analysis Platform (TCMSP, http://tcmspw.com/tcmsp.php) was used to extract all components of each CM included in this manuscript. The processes of absorption, distribution, metabolism, or elimination can affect pharmacodynamics and cause changes in drug bioavailability. Oral bioavailability was calculated using OBioavail1.1 [32] to filter out compounds that were not likely to be drugs. This software is based on a dataset of 805 structurally diverse drugs and drug-like molecules that have been critically evaluated for their oral bioavailability (%F) in humans. Three mathematical methods were applied to build various models: multiple linear regression (MLR), partial least square (PLS), and support vector machine (SVM) methods. The optimal model, using the SVM method, provides excellent performance with R^2^ = 0.80, SEE = 0.31 for the training set and Q2 = 0.72, SEP = 0.22 for the independent test set. In this study, compounds with OB ≥ 30% were selected as the threshold for analysis. The OB properties of all licorice compounds are also presented in the TcmSP™. The removal of non-drug-like compounds from the drug discovery lifecycle in the early stages can lead to tremendous resource savings. In this study, the Drug-likeness (DL) index in Eq. (1), using the Tanimoto coefficient [33], was computed for each licorice compound:

1 $$T({\text{x}},\;{\text{y}}) = \frac{{{\text{x}} \cdot {\text{y}}}}{{\left\| {\text{x}} \right\|^{2} + \left\| {\text{y}} \right\|^{2} - {\text{x}} \cdot {\text{y}}}}$$

where x represents the molecular properties of the licorice compound based on Dragon soft molecular descriptors, and y is the average molecular properties of all compounds in the DrugBank database (http://www.drugbank.ca/). A molecule that yields DL ≥ 0.18 is considered to be a ‘‘drug-like’’ compound and is selected as the candidate molecule for the following processes. The threshold of DL is determined based on the fact that the average DL index in DrugBank is 0.18. The drug-likeness indices of all licorice compounds are presented in TcmSP^TM^. Therefore, in our manuscript, we selected the components in each CM with OB ≥ 30% and DL index ≥ 0.18 as bioactive substances.

##### Identification of bioactive component-related genes for each CM

The genes of all substances in each CM were retrieved from the TCMSP database (http://tcmspw.com/tcmsp.php). Perl software was used to acquire a text file that included bioactive components (defined as OB ≥ 30% and DL index ≥ 0.18) and their related genes for each CM.

#### Screening of differential genes for osteoporosis and acquisition of the shared genes between bioactive ingredients of each CM and osteoporosis

##### Collecting genes for osteoporosis

The Gene expression omnibus (GEO) (https://www.ncbi.nlm.nih.gov/geo/), a public gene expression profile database of the National Center for Biotechnology Information, National Institutes of Health (USA), can be used to obtain a precise understanding of the molecular mechanisms underlying the onset of osteoporosis. In the current study, we collected osteoporosis-related gene expression profile chips by using “osteoporosis” as the search term in the high-throughput GEO database. After analyzing and comparing different chips, we selected the GSE35956 chip for analysis. This chip originated from the GPL570 [HG-U133_Plus_2] Affymetrix Human Genome U133 Plus 2.0 Array platform, which included five osteoporosis samples and five non-osteoporotic samples.

##### Collecting differential genes for osteoporosis

We used the limma package in R language to analyze differentially expressed genes identified in the GSE35956 chip. Subsequently, we filtered out upregulated and downregulated differentially expressed genes with |log2 fold change (FC)|> 1 and P < 0.05. To visualize the differentially expressed genes, the ggplot2 and pheatmap packages were used to draw volcano maps and heat maps.

##### Identification of shared genes between bioactive ingredients of each CM and osteoporosis

Bioactive ingredients of CMs shared common genes with osteoporosis. Perl software was used to acquire the shared genes.

#### Precise relationships among bioactive ingredients, shared genes, and pathways

In order to explore the pathways of shared genes between bioactive ingredients of each CM and osteoporosis, we installed the R packages (colorspace,” “stringi,” and “ggplot2), and to perform Kyoto Encyclopedia of Genes and Genomes (KEGG) enrichment analysis, bioconductor packages (DOSE,” “clusterProfiler,” and “enrichplot”) in the R software were installed. The species was set to “hsa,” and the filter values (P value and q-value) were set to 0.05. Subsequently, we manually summarized the precise relationships among bioactive ingredients, shared genes, and pathways.

### Python programming and software development

We used the Python programming language to write software source codes in Pycharm Community Edition 2021.2, for which we called six third-party libraries of Python (pandas, openpyxl, tkinter, tkinter.ttk, and ttkthemes and tkinter.messagebox libraries). We used pandas and openpyxl libraries to locate, read and retrieve data files; and tkinter, tkinter.ttk and ttkthemes libraries to write the overall interactive interface, to arrange various interactive elements such as input and output; further, we used tkinter.messagebox library combined with Python 3 basic syntax to create a trial-and-error mechanism. Finally, we generated a runnable Python file and used the pyinstaller library to package the Python file into an executable program.

### Software validation

We previously performed cell experiments, which were published in the Chinese Journal of Tissue Engineering Research in 2020 [13] to confirm the feasibility of our software. We also found several relevant articles [14, 31] to support the reliability of our software.

## Results

### Data mining—basic work for Python Programming

#### CM collection

**Fig. 2:**
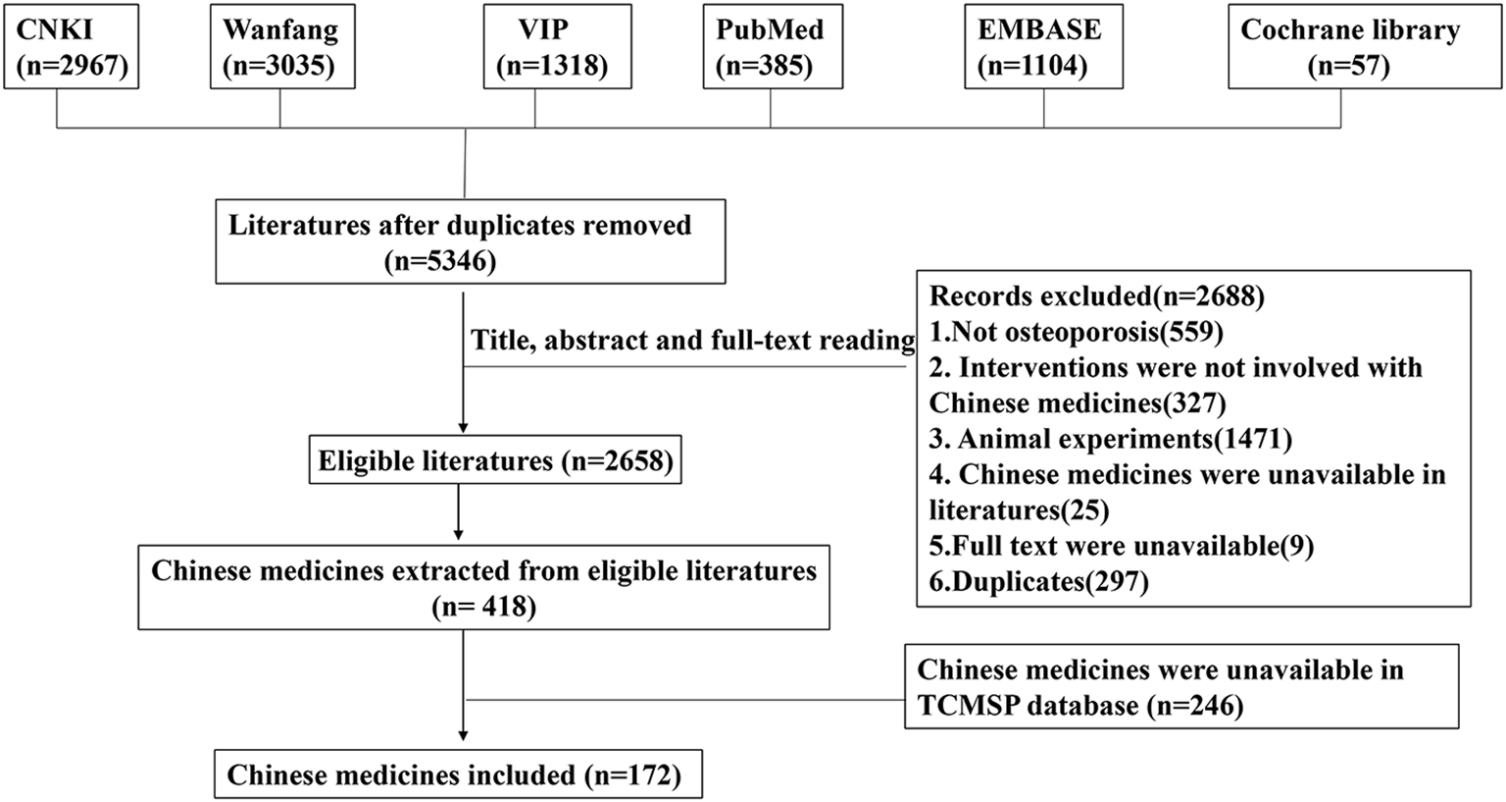
The detailed process of the literature selection and extraction of Chinese medicine

A preliminary search of the electronic databases retrieved 8866 articles. A total of 5346 articles remained after the deletion of duplicates using the NoteExpress software. Among these, 2688 articles were excluded based on the title, abstract, and full-text reading. A total of 2658 articles were left to extract data on CMs. We extracted 418 CMs from the eligible literature, of which 246 were unavailable in the TCMSP database. Therefore, a total of 172 CMs were included in our study (Fig. 2) and were also collated in an Excel file named ‘The list of Chinese medicines’ (Additional file 1).

Of the 172 CMs, 164 shared common genes with osteoporosis, with the exception of Aconiti Lateralis Radix Praeparata (fuzi), Borneolum Syntheticum (bingpian), Aconiti Radix (chuanwu), Rhizoma Dioscoreae Nipponicae (chuanshanlong), Dichroae Radix (changshan), Zanthoxylum nitidum (liangmianzhen), Trichosanthis Radix (tianhuafen), and Tetrapanacis Medulla (tongcao). Therefore, 164 CMs were used for the operations mentioned in the Methods section (Additional file 1). The total flavonoids of Drynariae Rhizoma have been used as a Chinese patent medicine (QiangGu Capsule) to treat osteoporosis in China. And Drynariae Rhizoma was top 1(41%; 1089 out of 2658) in the selection of eligible studies. Therefore, we used ‘Drynariae Rhizoma’ as a representative example to show the process in our manuscript.

#### Screening of bioactive ingredients and related genes for Drynariae Rhizoma

**Fig. 3:**
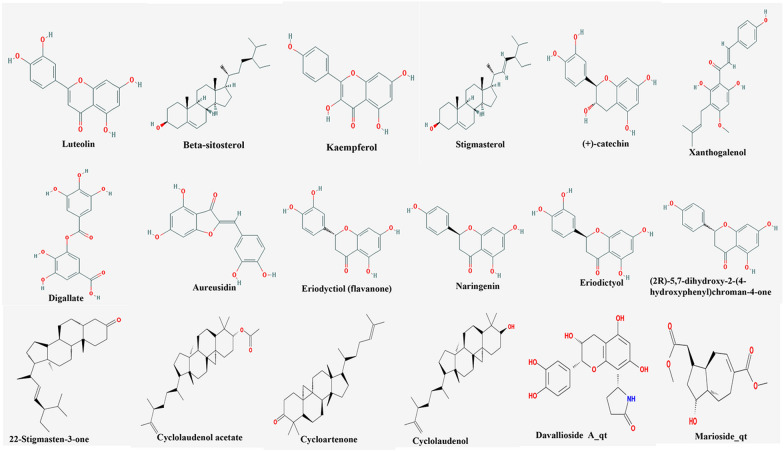
Molecular structures of 18 bioactive ingredients included in the study

After screening for bioactive ingredients OB ≥ 30% and DL ≥ 0.18 in the TCMSP database, ‘Drynariae Rhizoma’ was found to contain 18 bioactive ingredients (Table 1, Fig. 3). The genes were also predicted using the TCMSP database, and a total of 203 genes and 54 ingredients were obtained. Eventually, we obtained 15 bioactive ingredients and 164 genes using Perl software. Owing to the many-to-many relationship between bioactive ingredients and genes, a total of 296 corresponding relationships existed (Additional file 1). We have shown 25 of these corresponding relationships in Table 2.

**Table 1:** Details of the 18 bioactive ingredients in Drynariae Rhizoma

MolID	Molecule name	MW	Hdon	Hacc	OB (%)	BBB	DL	FASA-	HL
MOL001040	(2R)-5,7-dihydroxy-2-(4-hydroxyphenyl)chroman-4-one	272.27	3	5	42.3633211422	− 0.47578	0.21141	86.98999786376953	16.830309
MOL001978	Aureusidin	286.25	4	6	53.4232125103	− 0.52899	0.24465	111.12999725341797	21.022156
MOL002914	Eriodyctiol (flavanone)	288.27	4	6	41.3504271334	− 0.66394	0.2436	107.22000122070312	15.87634
MOL000449	Stigmasterol	412.77	1	1	43.8298515785	1.00045	0.75665	20.229999542236328	5.574595
MOL000358	Beta-sitosterol	414.79	1	1	36.9139058327	0.98588	0.75123	20.229999542236328	5.355491
MOL000422	Kaempferol	286.25	4	6	41.8822495352	− 0.55335	0.24066	111.12999725341797	14.743371
MOL004328	Naringenin	272.27	3	5	59.2938977347	− 0.37053	0.21128	86.98999786376953	16.976509
MOL000492	(+)-catechin	290.29	5	6	54.8264340523	− 0.72733	0.24164	110.37999725341797	0.609577
MOL005190	Eriodictyol	288.27	4	6	71.7926526045	− 0.54374	0.24372	107.22000122070312	15.81224
MOL000569	Digallate	322.24	6	9	61.8486180263	− 1.51806	0.25635	164.75	5.293312
MOL000006	Luteolin	286.25	4	6	36.1626293429	− 0.84349	0.24552	111.12999725341797	15.944492
MOL009061	22-Stigmasten-3-one	412.77	0	1	39.2536458582	1.28573	0.76134	17.06999969482422	4.628843
MOL009063	Cyclolaudenol acetate	482.87	0	2	41.6600704468	1.09348	0.78843	26.299999237060547	6.452029
MOL009075	Cycloartenone	424.78	0	1	40.5704663624	1.35192	0.7861	17.06999969482422	5.119279
MOL009076	Cyclolaudenol	440.83	1	1	39.0454111203	1.12385	0.78913	20.229999542236328	5.475195
MOL009078	Davallioside A_qt	373.39	6	8	62.6541727238	− 1.38824	0.50978	139.47999572753906	0.653298
MOL009087	Marioside_qt	296.4	1	5	70.7929483518	− 0.52681	0.19008	72.83000183105469	5.100967
MOL009091	Xanthogalenol	354.43	3	5	41.08185071	− 0.19032	0.31972	86.98999786	16.679284

*MolID* the ID number of bioactive ingredients in Drynariae Rhizoma; *Molecule name* the name of bioactive ingredient in Drynariae Rhizoma; *MW* molecular weight; *Hdon* hydrogen donor; *Hacc* hydrogen acceptor; *OB* oral bioavailability; *BBB* blood–brain barrier; *DL* drug-likeness; *FASA* fractional water accessible surface area of all atoms with negative partial charge; *HL* half-life

**Table 2:** Corresponding relationships between bioactive ingredients and genes

MolIdMolID	Bioactive ingredient in Drynariae Rhizoma	Bioactive ingredient-related gene
MOL000492	(+)-catechin	Beta-lactamase
MOL000492	(+)-catechin	Calmodulin
MOL000492	(+)-catechin	cAMP-dependent protein kinase catalytic subunit alpha
MOL000492	(+)-catechin	Estrogen receptor
MOL000492	(+)-catechin	Heat shock protein HSP 90-alpha
MOL000492	(+)-catechin	Hyaluronan synthase 2
MOL000492	(+)-catechin	Nuclear receptor coactivator 2
MOL000492	(+)-catechin	Prostaglandin G/H synthase 1
MOL000492	(+)-catechin	Prostaglandin G/H synthase 2
MOL000492	(+)-catechin	Retinoic acid receptor RXR-alpha
MOL001040	(2R)-5,7-dihydroxy-2-(4-hydroxyphenyl)chroman-4-one	Beta-lactamase
MOL001040	(2R)-5,7-dihydroxy-2-(4-hydroxyphenyl)chroman-4-one	cAMP-dependent protein kinase catalytic subunit alpha
MOL001040	(2R)-5,7-dihydroxy-2-(4-hydroxyphenyl)chroman-4-one	Estrogen receptor
MOL001040	(2R)-5,7-dihydroxy-2-(4-hydroxyphenyl)chroman-4-one	Glucocorticoid receptor
MOL001040	(2R)-5,7-dihydroxy-2-(4-hydroxyphenyl)chroman-4-one	Heat shock protein HSP 90-alpha
MOL001040	(2R)-5,7-dihydroxy-2-(4-hydroxyphenyl)chroman-4-one	Mineralocorticoid receptor
MOL001040	(2R)-5,7-dihydroxy-2-(4-hydroxyphenyl)chroman-4-one	Progesterone receptor
MOL001040	(2R)-5,7-dihydroxy-2-(4-hydroxyphenyl)chroman-4-one	Prostaglandin G/H synthase 1
MOL001040	(2R)-5,7-dihydroxy-2-(4-hydroxyphenyl)chroman-4-one	Prostaglandin G/H synthase 2
MOL009061	22-Stigmasten-3-one	Progesterone receptor
MOL001978	Aureusidin	Androgen receptor
MOL001978	Aureusidin	cAMP-dependent protein kinase catalytic subunit alpha
MOL001978	Aureusidin	Carbonic anhydrase 2
MOL001978	Aureusidin	Cell division protein kinase 2
MOL001978	Aureusidin	Cyclin-A2

*MolID* the ID number of the bioactive ingredients in Drynariae Rhizoma

#### Screening differential genes for osteoporosis and acquiring the shared genes between bioactive ingredients of Drynariae Rhizoma and osteoporosis

**Fig. 4:**
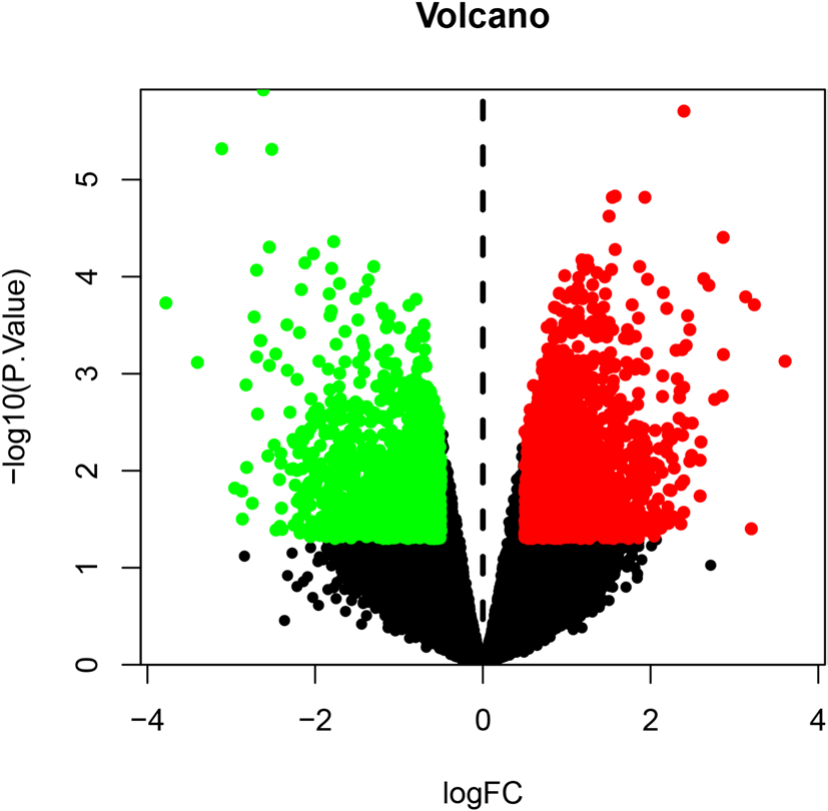
Gene volcano map for osteoporosis. Red represents upregulated genes, green represents downregulated genes, black represents no significant difference

**Fig. 5:**
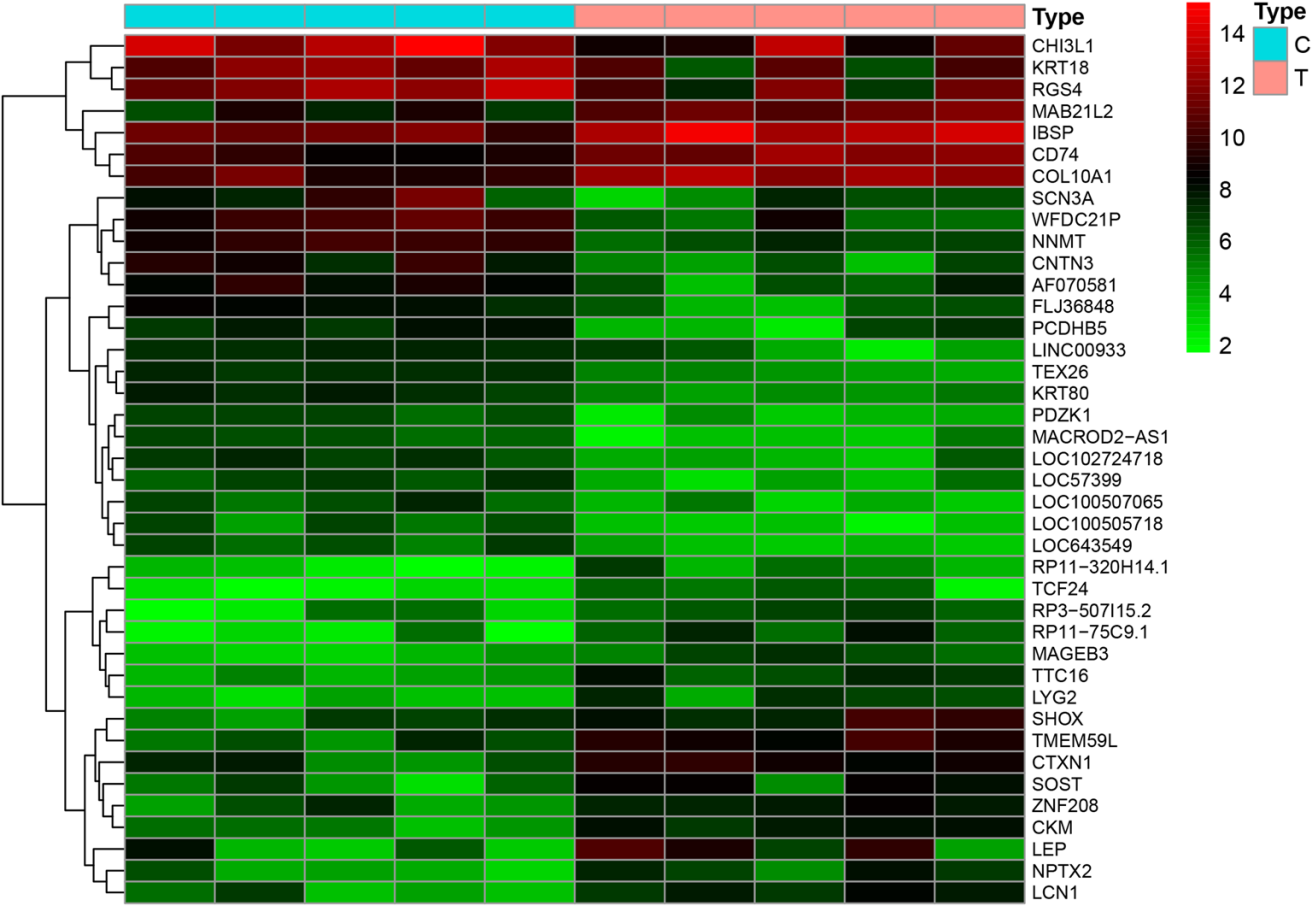
Gene heat map for osteoporosis. Red represents upregulated genes, green represents downregulated genes, black represent no significant difference. *C* non-osteoporotic controls; *T* patients with osteoporosis

We used the limma package to conduct differential gene expression analysis on osteoporosis data obtained from GSE35956. By comparing five osteoporosis samples with five non-osteoporotic samples in the GEO database, a total of 21,654 genes were identified. After screening for a P value < 0.05, and |log2 fold change (FC)|> 1, a total of 2789 genes were acquired (1465 upregulated genes and 1324 downregulated genes). As shown by the gene volcano and heat maps (Figs. 4, 5), the differential genes in the disease samples displayed a normal distribution.

We identified the shared genes between the bioactive ingredients of Drynariae Rhizoma and osteoporosis using Perl software. The results revealed 13 bioactive ingredients, 21 shared genes, and 50 corresponding relationships (Table 3).

**Table 3:** Corresponding relationship between bioactive ingredients in Drynariae Rhizoma and shared genes

MolID	Shared gene	Relationship
MOL001040	PTGS2	Target
MOL001040	PRKACA	Target
MOL001978	NOS2	Target
MOL001978	PTGS2	Target
MOL001978	PRKACA	Target
MOL001978	CCNA2	Target
MOL002914	PTGS2	Target
MOL002914	PRKACA	Target
MOL000449	PTGS2	Target
MOL000449	ADRA2A	Target
MOL000449	PRKACA	Target
MOL000358	PTGS2	Target
MOL000358	PRKACA	Target
MOL000422	NOS2	Target
MOL000422	PTGS2	Target
MOL000422	PRKACA	Target
MOL000422	TOP2A	Target
MOL000422	RELA	Target
MOL000422	AHSA1	Target
MOL000422	CDK1	Target
MOL000422	ICAM1	Target
MOL000422	AHR	Target
MOL000422	GSTM1	Target
MOL000422	GSTM2	Target
MOL004328	PTGS2	Target
MOL004328	PRKACA	Target
MOL004328	RELA	Target
MOL004328	LDLR	Target
MOL004328	SOAT2	Target
MOL004328	ABAT	Target
MOL000492	PTGS2	Target
MOL000492	PRKACA	Target
MOL005190	PTGS2	Target
MOL005190	PRKACA	Target
MOL000569	PTGS2	Target
MOL000006	PTGS2	Target
MOL000006	PRKACA	Target
MOL000006	RELA	Target
MOL000006	CDKN1A	Target
MOL000006	TOP1	Target
MOL000006	ICAM1	Target
MOL000006	BIRC5	Target
MOL000006	CCNB1	Target
MOL000006	TOP2A	Target
MOL000006	NUF2	Target
MOL009078	PTGS2	Target
MOL009078	TOP2A	Target
MOL009091	NOS2	Target
MOL009091	PTGS2	Target
MOL009091	CCNA2	Target

*MolID* the ID number of bioactive ingredients in Drynariae Rhizoma; *Shared gene* the shared gene between bioactive ingredients of Drynariae Rhizoma and osteoporosis

#### Precise relationships among bioactive ingredients, shared genes and pathways

**Fig. 6:**
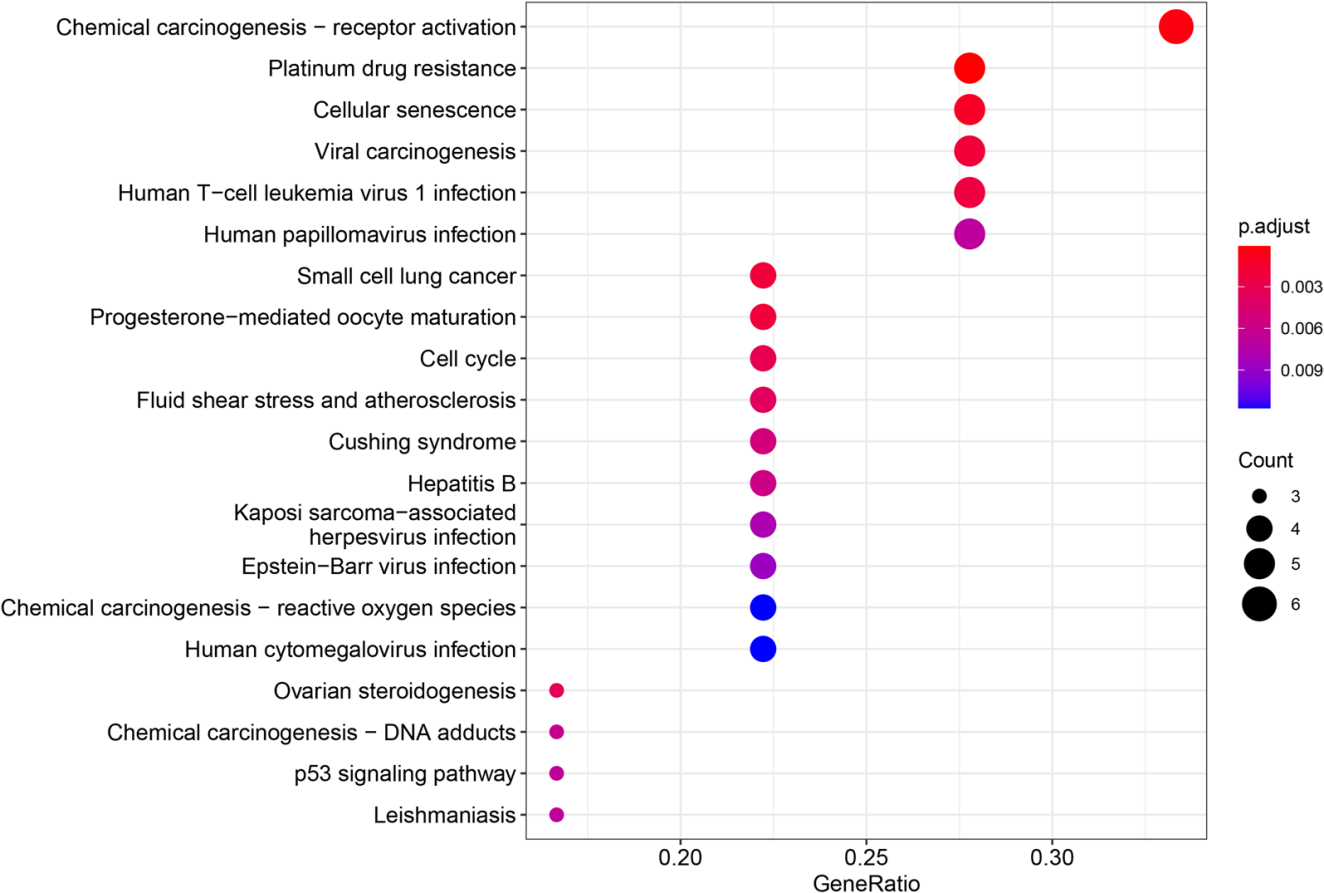
KEGG bubble. The horizontal axis represents the gene proportion enriched in each entry, the vertical axis represents the enrichment degree based on the corrected P value

**Fig. 7:**
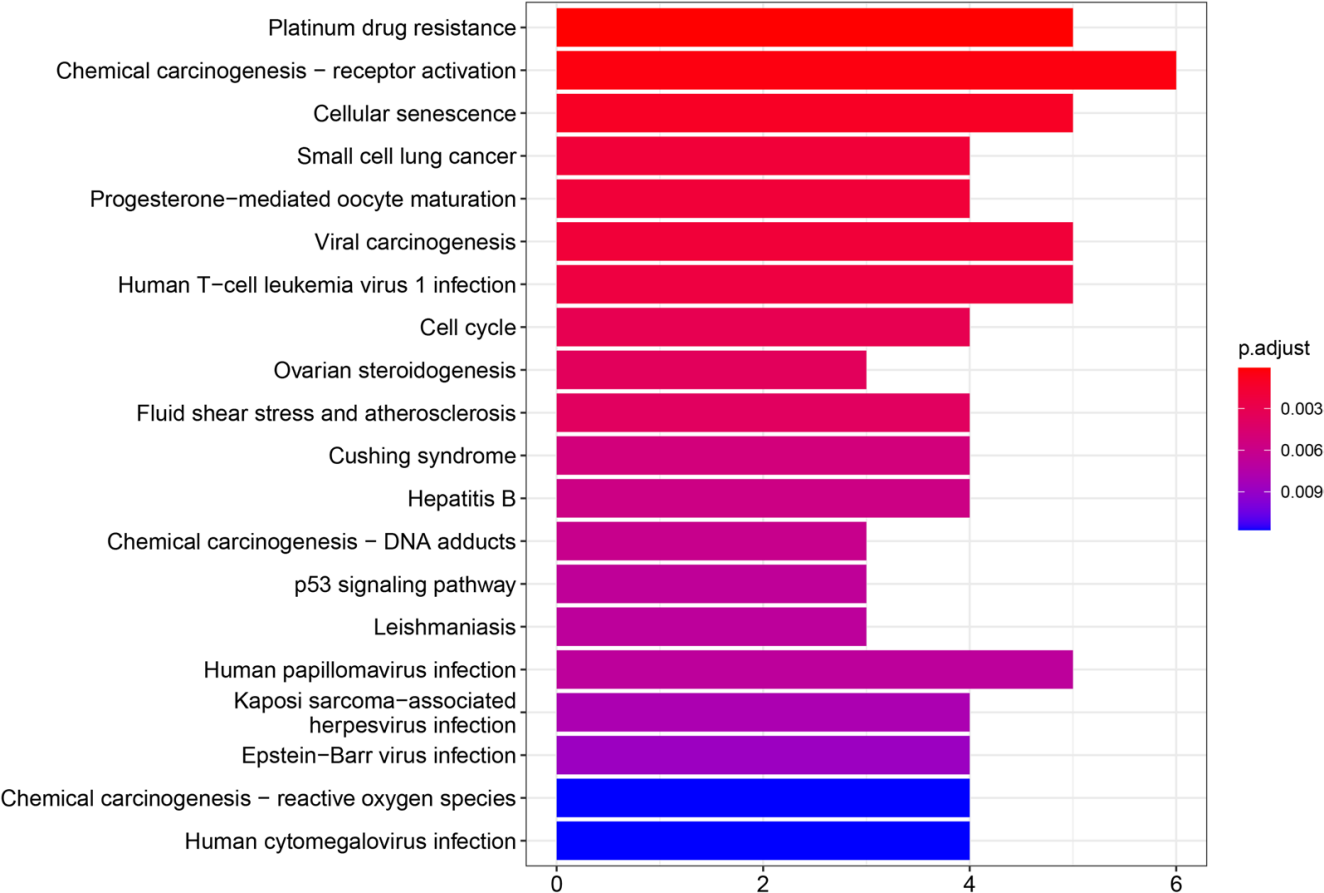
KEGG barplot. The horizontal axis represents the number of genes enriched in each item, the color representing the enrichment significance based on the corrected P value

KEGG pathway analysis of shared genes was conducted to explore the pathways of Drynariae Rhizoma in osteoporosis. According to the KEGG enrichment results, the involved pathways included chemical carcinogenesis, receptor activation, platinum drug resistance, cellular senescence, viral carcinogenesis, human T-cell leukemia virus 1 infection, small cell lung cancer, progesterone-mediated oocyte maturation, cell cycle, fluid shear stress and atherosclerosis, Cushing syndrome, and hepatitis B (Figs. 6, 7). We further investigated the precise relationships among bioactive ingredients, shared genes, and pathways. We have shown 22 of the 44 precise relationships in our manuscript (Table 4).

**Table 4:** The precise relationships among bioactive ingredients, shared genes, and pathways

Molecule name	Gene name	Pathway name	Pathway name	Pathway name	Pathway name	Pathway name	Pathway name	Pathway name	Pathway name
Luteolin	Intercellular adhesion molecule 1 (CD54), human rhinovirus receptor	Human T-cell leukemia virus 1 infection	Kaposi sarcoma-associated herpesvirus infection	Epstein-Barr virus infection	Fluid shear stress and atherosclerosis				
Luteolin	Baculoviral IAP repeat-containing 5 (surviving)	Platinum drug resistance	Hepatitis B	Chemical carcinogenesis—receptor activation					
Luteolin	Cyclin B1	Cell cycle	p53 signaling pathway	Cellular senescence	Progesterone-mediated oocyte maturation				
Luteolin	Topoisomerase (DNA) II alpha 170 kda	Platinum drug resistance							
Beta-sitosterol	Prostaglandin-endoperoxide synthase 2 (prostaglandin G/H synthase and cyclooxygenase)	Ovarian steroidogenesis	Leishmaniasis	Human cytomegalovirus infection	Human papillomavirus infection	Kaposi sarcoma-associated herpesvirus infection	Chemical carcinogenesis—DNA adducts	Small cell lung cancer	
Beta-sitosterol	Protein kinase, camp-dependent, catalytic, alpha	Ovarian steroidogenesis	Progesterone-mediated oocyte maturation	Cushing syndrome	Human cytomegalovirus infection	Human papillomavirus infection	Human T-cell leukemia virus 1 infection	Viral carcinogenesis	Chemical carcinogenesis—receptor activation
Kaempferol	Nitric oxide synthase 2	Leishmaniasis	Small cell lung cancer						
Kaempferol	Prostaglandin-endoperoxide synthase 2 (prostaglandin G/H synthase and cyclooxygenase)	Ovarian steroidogenesis	Leishmaniasis	Human cytomegalovirus infection	Human papillomavirus infection	Kaposi sarcoma-associated herpesvirus infection	Chemical carcinogenesis—DNA adducts	Small cell lung cancer	
Kaempferol	Protein kinase, camp-dependent, catalytic, alpha	Ovarian steroidogenesis	Progesterone-mediated oocyte maturation	Cushing syndrome	Human cytomegalovirus infection	Human papillomavirus infection	Human T-cell leukemia virus 1 infection	Viral carcinogenesis	Chemical carcinogenesis—receptor activation
Kaempferol	Topoisomerase (DNA) II alpha 170 kda	Platinum drug resistance							
Kaempferol	Cyclin-dependent kinase 1	Cell cycle	p53 signaling pathway	Cellular senescence	Progesterone-mediated oocyte maturation	Viral carcinogenesis			
Kaempferol	Intercellular adhesion molecule 1 (CD54), human rhinovirus receptor	Human T-cell leukemia virus 1 infection	Kaposi sarcoma-associated herpesvirus infection	Epstein-Barr virus infection	Fluid shear stress and atherosclerosis				
Kaempferol	Aryl hydrocarbon receptor	Cushing syndrome	Chemical carcinogenesis—receptor activation	Chemical carcinogenesis—reactive oxygen species					
Kaempferol	Glutathione S-transferase M1	Platinum drug resistance	Chemical carcinogenesis—DNA adducts	Chemical carcinogenesis—receptor activation	Chemical carcinogenesis—reactive oxygen species	Fluid shear stress and atherosclerosis			
Kaempferol	Glutathione S-transferase M2	Platinum drug resistance	Chemical carcinogenesis—DNA adducts	Chemical carcinogenesis—receptor activation	Chemical carcinogenesis—reactive oxygen species	Fluid shear stress and atherosclerosis			
Stigmasterol	Prostaglandin-endoperoxide synthase 2 (prostaglandin G/H synthase and cyclooxygenase)	Ovarian steroidogenesis	Leishmaniasis	Human cytomegalovirus infection	Human papillomavirus infection	Kaposi sarcoma-associated herpesvirus infection	Chemical carcinogenesis—DNA adducts	Small cell lung cancer	
Stigmasterol	Protein kinase, camp-dependent, catalytic, alpha	Ovarian steroidogenesis	Progesterone-mediated oocyte maturation	Cushing syndrome	Human cytomegalovirus infection	Human papillomavirus infection	Human T-cell leukemia virus 1 infection	Viral carcinogenesis	Chemical carcinogenesis—receptor activation
(+)-Catechin	Prostaglandin-endoperoxide synthase 2 (prostaglandin G/H synthase and cyclooxygenase)	Ovarian steroidogenesis	Leishmaniasis	Human cytomegalovirus infection	Human papillomavirus infection	Kaposi sarcoma-associated herpesvirus infection	Chemical carcinogenesis—DNA adducts	Small cell lung cancer	
(+)-Catechin	Protein kinase, camp-dependent, catalytic, alpha	Ovarian steroidogenesis	Progesterone-mediated oocyte maturation	Cushing syndrome	Human cytomegalovirus infection	Human papillomavirus infection	Human T-cell leukemia virus 1 infection	Viral carcinogenesis	Chemical carcinogenesis—receptor activation
Digallate	Prostaglandin-endoperoxide synthase 2 (prostaglandin G/H synthase and cyclooxygenase)	Ovarian steroidogenesis	Leishmaniasis	Human cytomegalovirus infection	Human papillomavirus infection	Kaposi sarcoma-associated herpesvirus infection	Chemical carcinogenesis—DNA adducts	Small cell lung cancer	
(2R)-5,7-dihydroxy-2-(4-hydroxyphenyl)chroman-4-one	Prostaglandin-endoperoxide synthase 2 (prostaglandin G/H synthase and cyclooxygenase)	Ovarian steroidogenesis	Leishmaniasis	Human cytomegalovirus infection	Human papillomavirus infection	Kaposi sarcoma-associated herpesvirus infection	Chemical carcinogenesis—DNA adducts	Small cell lung cancer	
Aureusidin	Nitric oxide synthase 2	Leishmaniasis	Small cell lung cancer						

*Molecule name* the name of bioactive ingredient

### Python programming—developing a software

#### Step 1. Creating a Python file

We installed Pycharm Community Edition 2021.2 software and created a Python file for the host code.

#### Step 2. Importing all required third-party libraries

We used six libraries in Python to support the programming. We installed the required third-party libraries in Pycharm Community Edition 2021.2 software, and imported them into the coding page of the Python file as follows:

import tkinter.messagebox

from tkinter import *

import pandas as pd

from tkinter.ttk import *

from ttkthemes import *

import openpyxl

#### Step 3. Creating an interactive interface

We used the previously imported tkinter, tkinter.ttk, and ttkthemes libraries to create an interactive interface that included the user input side, search, user close command button, and text output box. Among the three imported libraries, the tkinter library was used to create the interface program; tkinter.ttk and ttk.theme libraries were used to identify the interface. The detailed code was as follows:
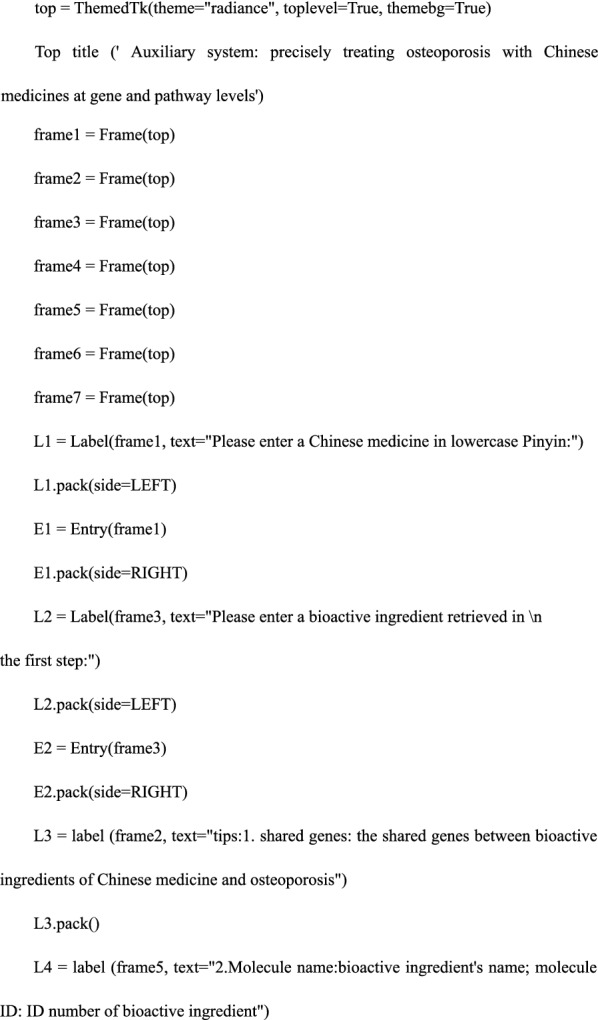

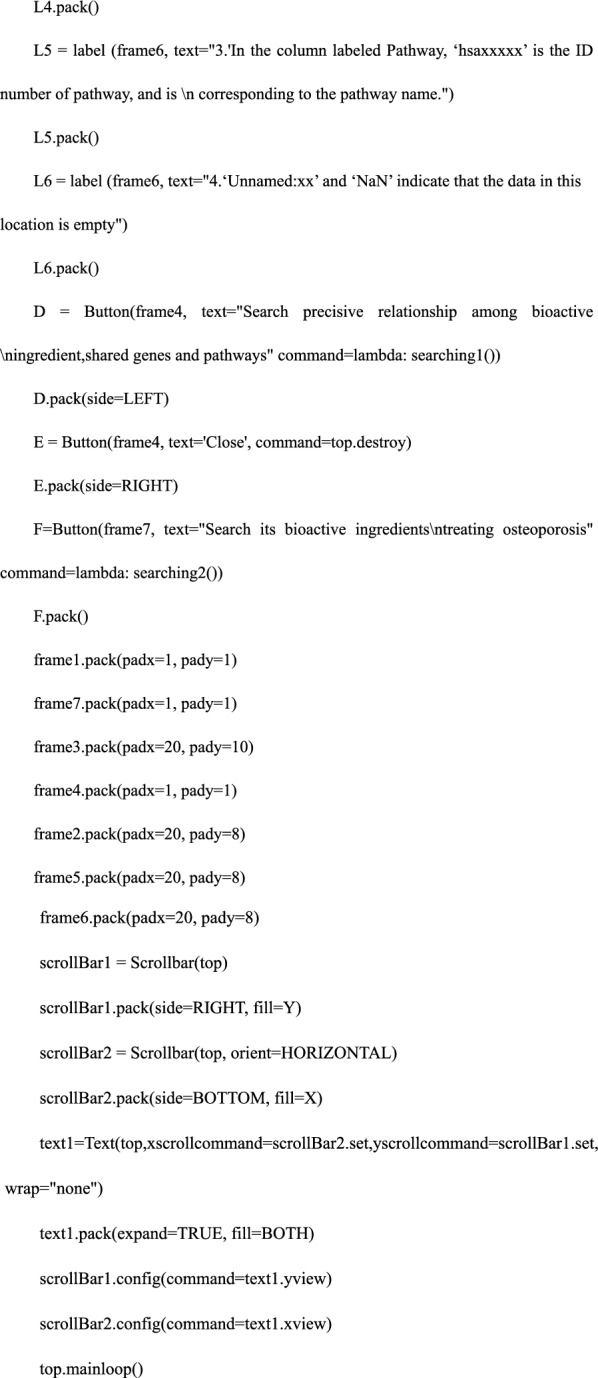


#### Step 4. Defining the search functions—the core of the software

We defined the search functions and constructed; searching 1: input Chinese medicine in lowercase Pinyin—output the bioactive ingredients treating osteoporosis; searching 2: input one bioactive ingredient obtained in searching 1—output the precise relationship among bioactive ingredients, shared genes, and pathways. In this process, we used pandas and openpyxl libraries to locate, read and retrieve data files; we used the “try… Except” function of Python and tkinter.messagebox library to create a trial-and-error mechanism. The detailed code is as follows:
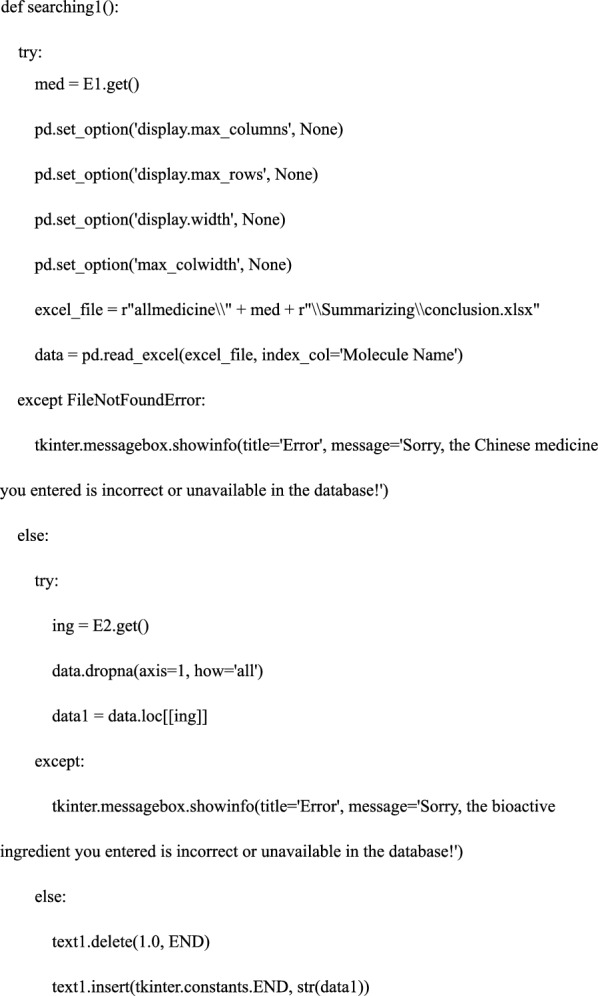

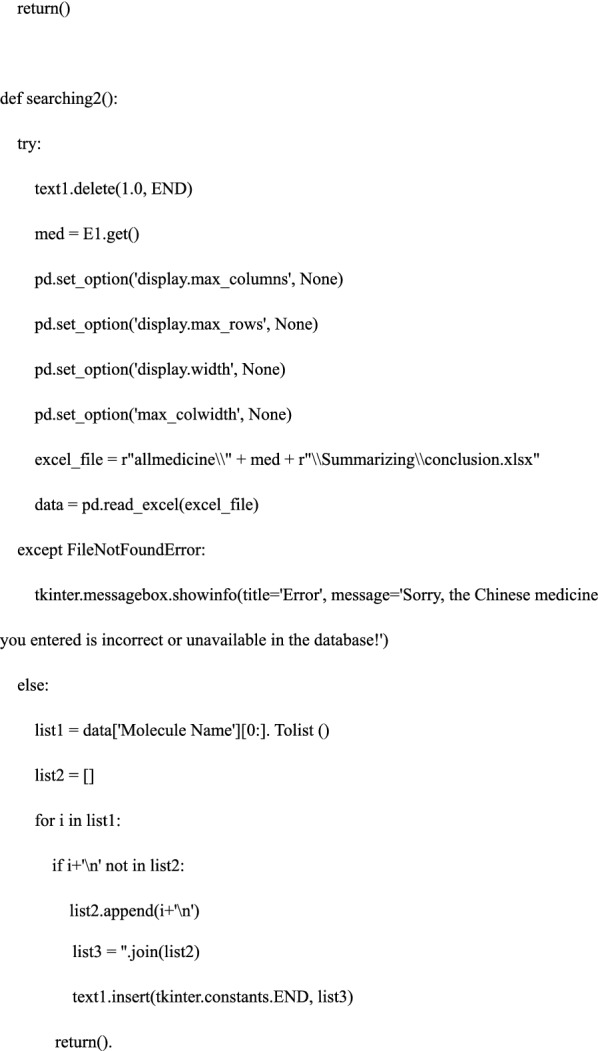


#### Step 5. Forming an executable program file

**Fig. 8:**
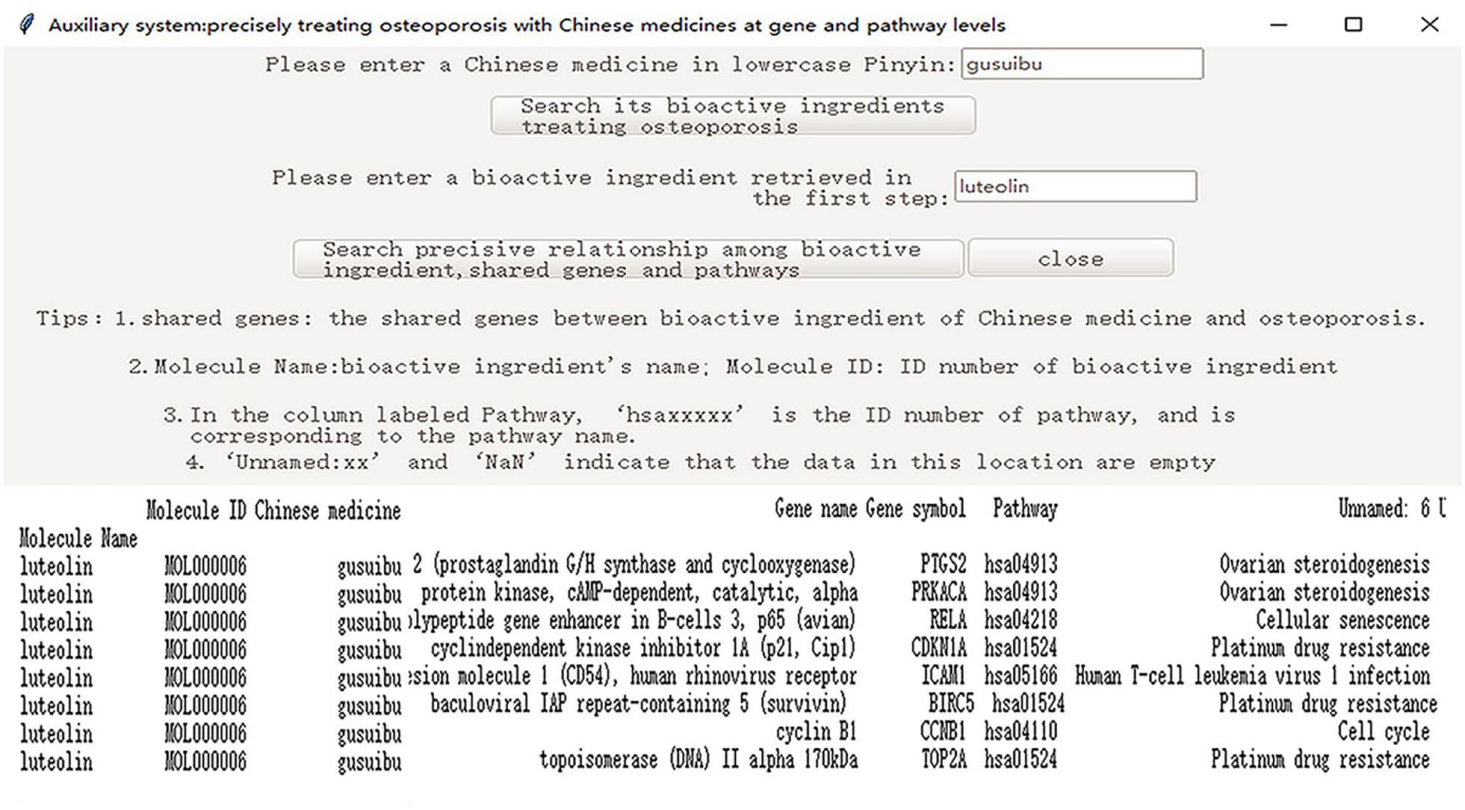
The presentation of the software’s functions

To run the software successfully on different computers, the Pyinstaller library was used to create an executable program file by packaging the codes of the Python file. We used Rhizoma Drynariae (gusuibu) as an example to present the functions of the executable program file software as follows (Fig. 8, Additional file 2).

### Software validation

**Fig. 9:**
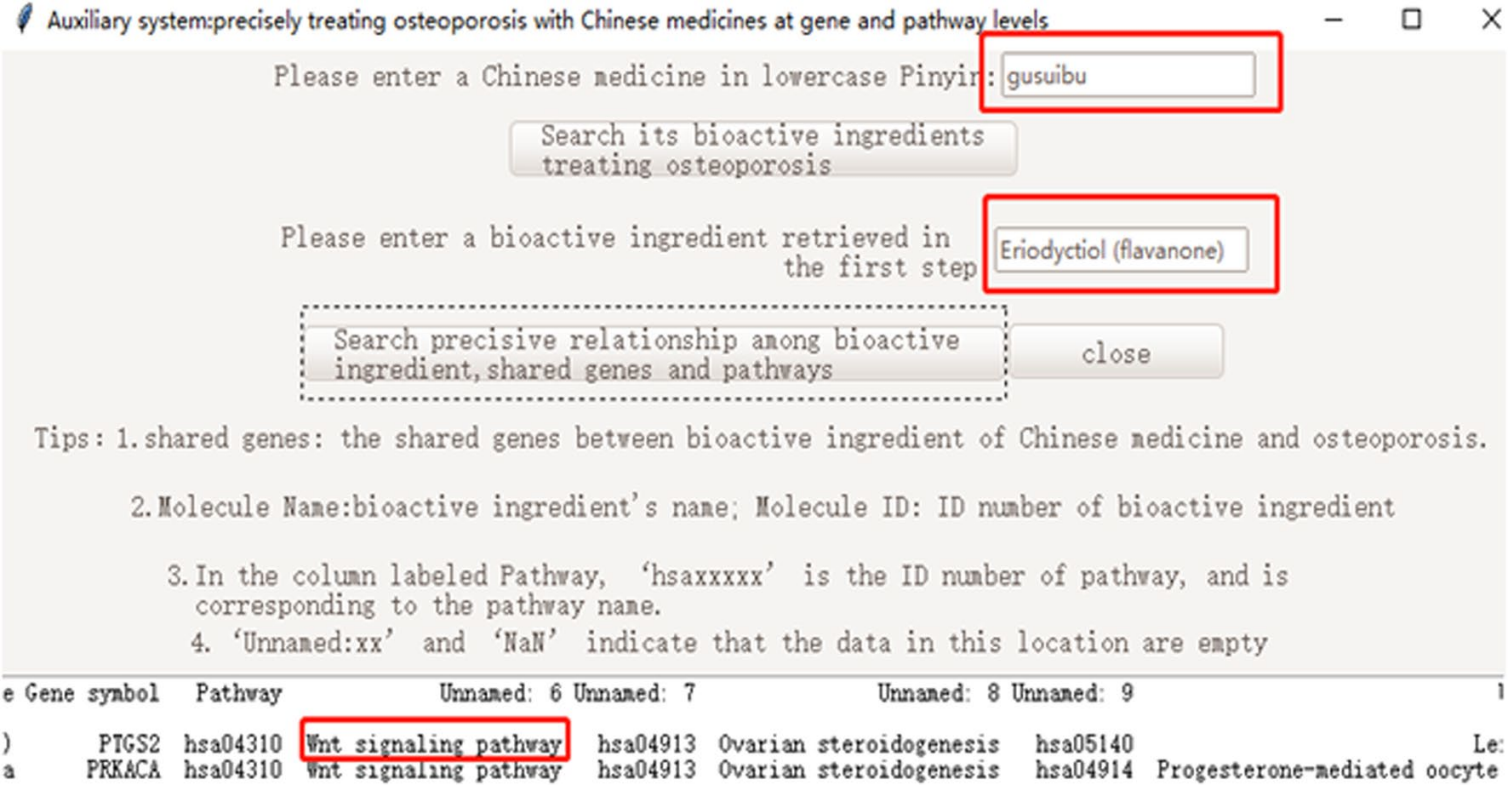
The presentation of flavonoids of Rhizoma Drynariae treating osteoporosis via Wnt signaling pathway

Our software showed that the flavone Rhizoma Drynariae (gusuibu in lowercase pinyin) might treat osteoporosis via the Wnt signaling pathway (Fig. 9). Li et al. [14] reported that the total flavonoids of Rhizoma Drynariae could promote differentiation of osteoblasts and growth of bone graft in an induced membrane, partly by activating the Wnt/β-Catenin signaling pathway [14]. Data from our cell experiment published in the Chinese Journal of Tissue Engineering Research [13] also supported the result of our software, and was outlined below.

### Data from our cell experiment

#### Materials

Mouse MC3T3-E1 osteoblast line was provided from Peking Union Medical College, Beijing, China; Rhizoma Drynariae total flavonoids were provided from Beijing Qihuang Pharmaceutical Co., Ltd.

#### Groups

- ① Normal group.
- ② DKK1 group: Wnt pathway inhibitor DKK1 (0.1 mg/L) blocked the Wnt/β-catenin signaling pathway;
- ③ DKK1 + transforming growth factor β (10 μg/L) group;
- ④ DKK1 + total flavonoids of Rhizoma Drynariae (100 mg/L) group;
- ⑤ DKK1 + total flavonoids of Rhizoma Drynariae (250 mg/L) group;

The cells were harvested at both 24 and 48 h of treatment.

##### Real-time PCR analysis

**Fig. 10:**
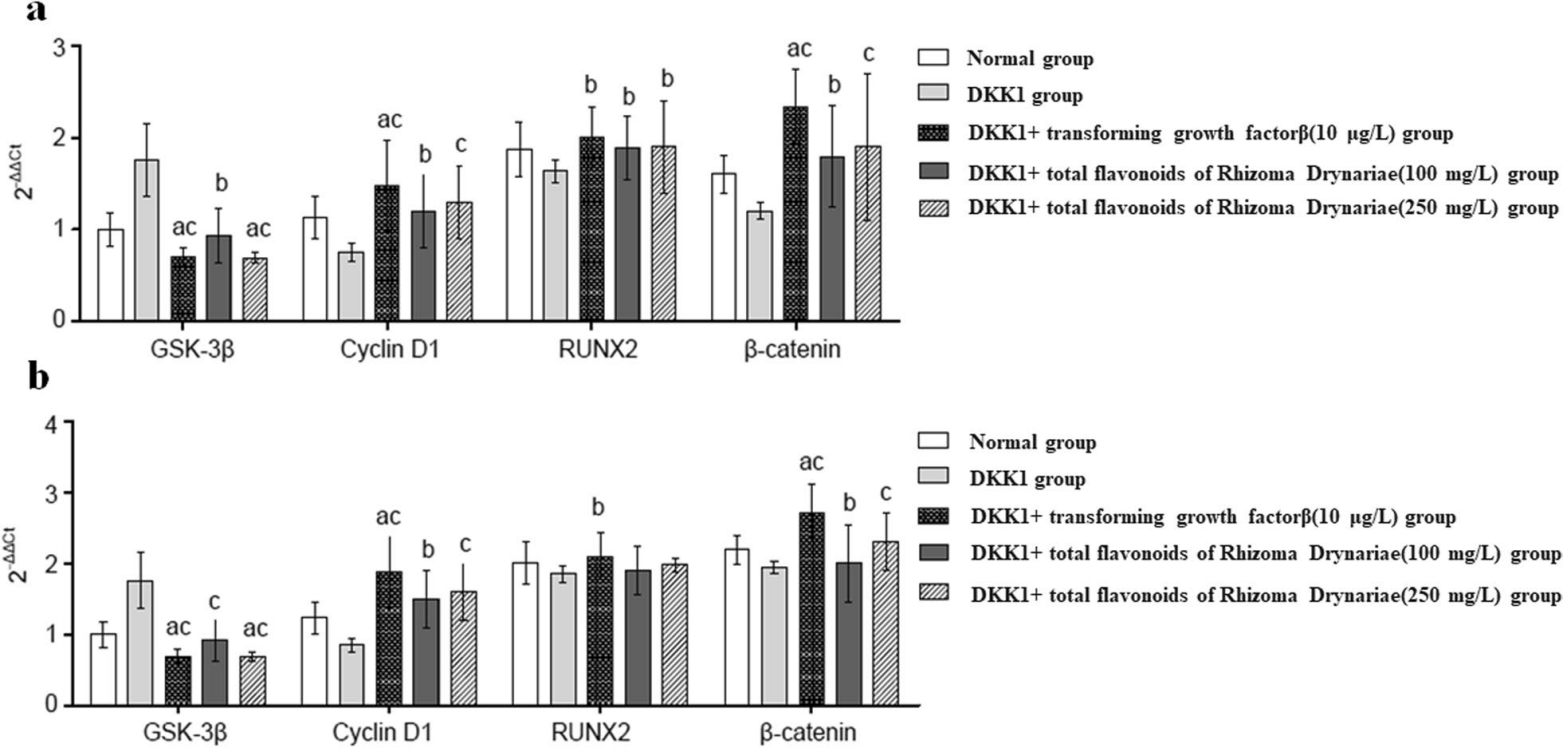
Expression of related genes in MC3T3-E1 cells of each group after 24 h (**a**) and 48 h (**b**) of treatment, compared with normal group, ^a^P < 0.01; compared with DKK1 group, ^b^P < 0.05, ^c^P < 0.01

Compared with the DKK1 group, the DKK1 + transforming growth factor β group, and the DKK1 + total flavonoids of Rhizoma Drynariae (100 mg/L, 250 mg/L) groups had a higher mRNA expression of β-catenin, RUNX2 and Cyclin D1 (P < 0.05), and had a lower mRNA expression of GSK-3β (P < 0.05) after 24 h of treatment (Fig. 10a).

**Fig. 11:**
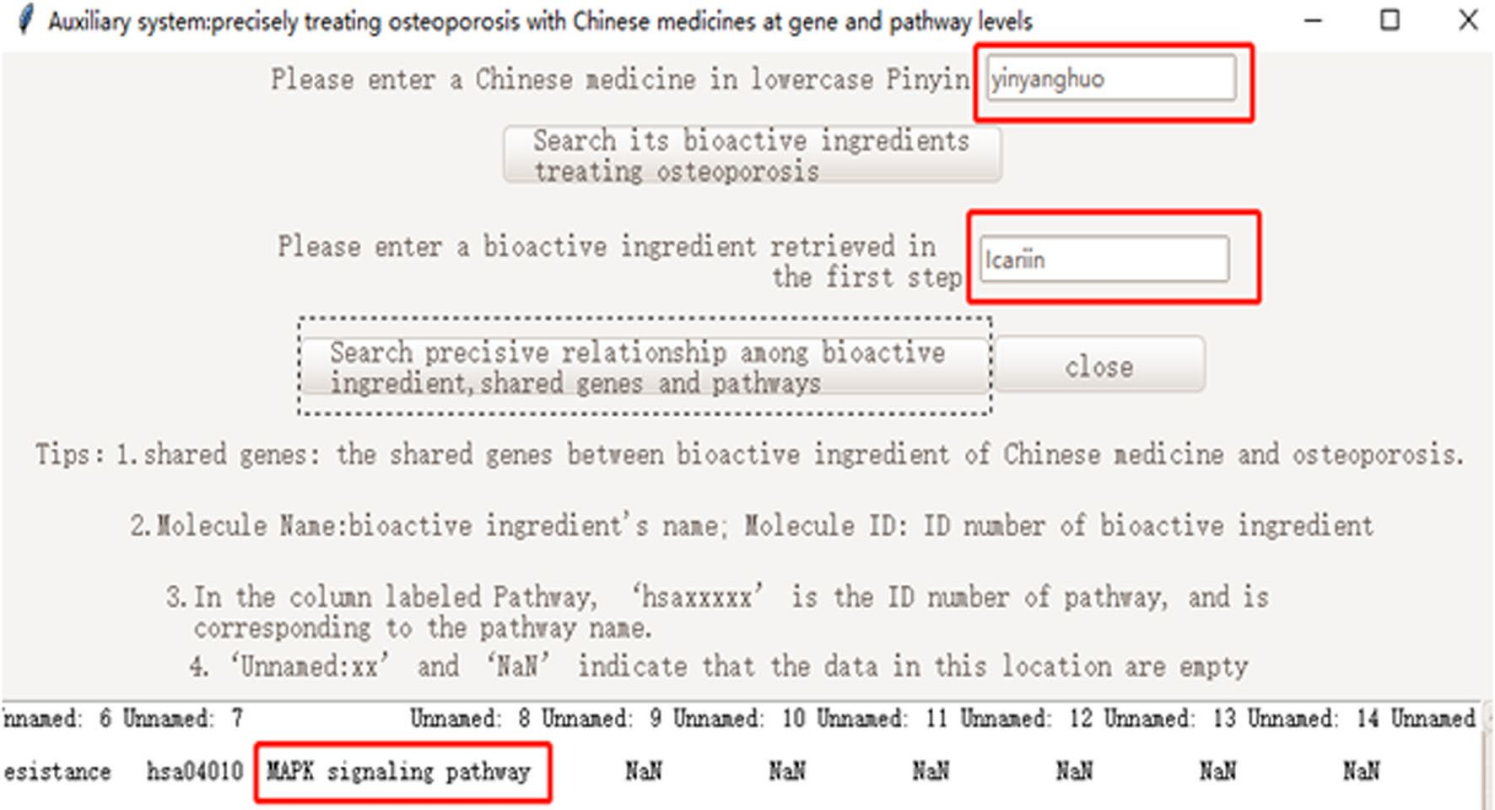
The presentation of icariin in Epimedium treating osteoporosis via MAPK signaling pathway

The results of our software also showed that icariin, from Epimedium (yinyanghuo in lowercase pinyin), might treat osteoporosis via the MAPK signaling pathway (Fig. 11). Wu et al. reported that icariin, from Epimedium, could induce osteogenic differentiation of bone mesenchymal stem cells via the MAPK signaling pathway [31]. These results supported the application of our software.

## Discussion

Osteoporosis, the most common chronic metabolic bone disease, is characterized by low bone mass and microarchitectural deterioration of bone tissue. Osteoporosis can enhance bone fragility and increase the risk of fractures [5]. It has been estimated that more than 200 million men and women suffer from osteoporosis worldwide [20]. With the aging population, osteoporosis is becoming an increasingly significant public health problem. We used the limma package to conduct differential gene expression analysis on osteoporosis data obtained from GSE35956. The results showed that a total of 2789 genes were acquired, including 1465 upregulated genes and 1324 downregulated genes.

TCM can promote bone formation via osteogenesis of MSCs and osteoblasts [8]. In Korean traditional medicine, the seeds of Carthami Flos (Hong-Hua) are used to promote bone formation and prevent osteoporosis. To support this use, a previous study showed that the defatted seeds of Carthamus tinctorius could protect ovariectomized rats from trabecular bone loss [11]. Aqueous cistanches extract improved bone mineral density, bone mineral content, and bone biomechanical indices (maximum load and displacement at maximum load) in ovariectomized rats in a dose dependent manner [15]. Icariin, a chemical constituent of Epimedium, has been reported to promote bone health [12, 18, 34]. Animal experiments have demonstrated that icariin is involved in bone mesenchymal stem cell differentiation and is also involved in the secretion of early osteoblast differentiation factors, such as osteocalcin [2]. After searching six databases (PubMed, EMBASE, Cochrane library, CNKI, VIP, and Wanfang databases), we finally included 164 CMs in our manuscript.

Precision medicine aims to maximize the therapeutic effectiveness by considering individual differences in genes, environment, and lifestyle [10]. We are at an accelerating point in the ‘precision medicine’-based research, driven by advances in molecular genomics, computational speed, and bioinformatics [7]. Notably, the field of oncology has been transformed by precision medicine; for example, tumors of metastatic breast cancer expressing human epidermal growth factor receptor 2 (EGFR2) have been proven to benefit from the EGFR2 monoclonal antibody trastuzumab [26]. Under these conditions, we screened and acquired bioactive ingredients and related genes for each CM using the TCMSP database. We screened differential genes for osteoporosis using the GEO database and acquired the shared genes between bioactive ingredients of each CM and osteoporosis using Perl software. We explored the pathways of shared genes in osteoporosis for each CM by KEGG pathway analysis. Finally, we acquired the precise relationships among bioactive ingredients, shared genes, and pathways.

As precision medicine moves forward, new strategies require carriers to express them. In this study, we successfully created an executable program file to achieve precise treatment of osteoporosis using CMs at the gene and pathway levels, and supported the reliability and facticity of our software by our experimental data [13] and several published articles [14, 31].

## Conclusions

Our study showed that the combination of data mining and Python programming could be applied to design software to achieve precise treatment of osteoporosis with CMs at the gene and pathway levels. The results of our study demonstrated that to some extent, this executable program file may achieve precision treatment of CMs for osteoporosis, and may unveil the biochemical basis and underlying mechanisms of CMs for treating osteoporosis. Our previously published study [13] and several published articles [14, 31] found that the total flavonoids of Rhizoma Drynariae and icariin of Epimedium might treat osteoporosis via the Wnt and MAPK signaling pathways, respectively, which successfully support the application of our software. Further experimental verification of the results predicted by our software is required to develop precision TCM with clinical translational potential in the future.

### Supplementary Information

**Additional file 1. **The contents of data mining.

**Additional file 2. **The executable program file software.

## Data Availability

The data and materials in this study are available from the corresponding author upon request.

## Appendix 1

#1 Osteoporosis [MeSH]

#2 Osteoporoses [Title/Abstract]

#3 Osteoporosis, Post-Traumatic [Title/Abstract]

#4 Osteoporosis, Post Traumatic [Title/Abstract]

#5 Post-Traumatic Osteoporoses [Title/Abstract]

#6 Post-Traumatic Osteoporosis [Title/Abstract]

#7 Osteoporosis, Senile [Title/Abstract]

#8 Osteoporoses, Senile [Title/Abstract]

#9 Senile Osteoporoses [Title/Abstract]

#10 Osteoporosis, Involutional [Title/Abstract]

#11 Senile Osteoporosis [Title/Abstract]

#12 Osteoporosis, Age-Related [Title/Abstract]

#13 Osteoporosis, Age Related [Title/Abstract]

#14 Bone Loss, Age-Related [Title/Abstract]

#15 Age-Related Bone Loss [Title/Abstract]

#16 Age-Related Bone Losses [Title/Abstract]

#17 Bone Loss, Age Related [Title/Abstract]

#18 Bone Losses, Age-Related [Title/Abstract]

#19 Age-Related Osteoporosis [Title/Abstract]

#20 Age Related Osteoporosis [Title/Abstract]

#21 Age-Related Osteoporoses [Title/Abstract]

#22 Osteoporoses, Age-Related [Title/Abstract]

#23#1OR#2OR#3OR#4OR#5OR#6OR#7OR#8OR#9OR#10OR#11OR#12OR#13OR#14OR#15OR#16OR#17OR#18OR#19OR#20OR#21OR#22

#24 traditional Chinese medicine

#25 decoction

#26 formula

#27 herbal medicine

#28 herbs

#29#24OR#25OR#26OR#27OR#28

#30#23AND#29

## Abbreviations

OP: Osteoporosis
CMs: Chinese medicines
CNKI: China national knowledge infrastructure
GEO: Gene expression omnibus
VIP database: China science and technology journal database
TCMSP: Traditional Chinese medicine systems pharmacology
BMD: Bone mineral density
TCM: Traditional Chinese medicine
MSCs: Mesenchymal stem cells
ADME: Absorption, distribution, metabolism, excretion
DL: Drug-likeness
OB: Oral bioavailability
KEGG: Kyoto Encyclopedia of Genes and Genomes
EGFR2: Epidermal growth factor receptor 2
siRNA: Small interfering RNA
NFATc1: Nuclear factor of active T cells
Runx2: Runx related transcription factor 2
MLR: Multiple linear regression
PLS: Partial least square
SVM: Support vector machine
